# Relationship between myocardial enzyme levels, hepatic function and metabolic acidosis in children with rotavirus infection diarrhea

**DOI:** 10.12669/pjms.36.6.2325

**Published:** 2020

**Authors:** Na-ying Zuo, Yuan-da Zhang, Qing-wei Dong, Li-po Han

**Affiliations:** 1 Na-ying Zuo, Department of Gastroenterology, Baoding Children’s Hospital, Baoding, Hebei, 071000, P.R. China; Key Laborary of Clinical Research on Respiratory Digestive Disease, Hebei Baoding, 071000, China; 2 Yuan-da Zhang, Department of Gastroenterology, Baoding Children’s Hospital, Baoding, Hebei, 071000, P.R. China; Key Laborary of Clinical Research on Respiratory Digestive Disease, Hebei Baoding, 071000, China; 3 Qing-wei Dong, Department of Gastroenterology, Baoding Children’s Hospital, Baoding, Hebei, 071000, P.R. China; Key Laborary of Clinical Research on Respiratory Digestive Disease, Hebei Baoding, 071000, China; 4 Li-po Han, Dept. of Ophthalmology, Baoding Children’s Hospital, Baoding, Hebei, 071000, P.R. China

**Keywords:** Acidosis, Hepatic function, Infectious diarrhea, Myocardial enzyme, Rotavirus

## Abstract

**Objective:**

To investigate the relationship between myocardial enzymes, liver function and metabolic acidosis in children with rotavirus infection diarrhea.

**Methods:**

The data of 70 children with infectious diarrhea treated in Baoding Children’s Hospital, China, from October 2017 to April 2018 were retrospectively studied. The antigen of rotavirus in feces was positive by colloidal gold method. According to the clinical features of biochemical indicators and mental status, the patients were divided into four groups, an acidosis-free group, a mild acidosis group, a moderate acidosis group and a severe acidosis group, in line with acidosis severity. In addition to detecting the hepatic functions of the pediatric patients in the four groups, including aspartate aminotransferase (AST), alanine aminotransfer (ALT) levels, and myocardial enzyme levels (e.g., creatine kinase, or CK, and creatine kinase isoenzyme, or CK-MB), the relationships of hepatic function, myocardial enzyme levels and acidosis severity of the patients with infectious diarrhea caused by rotavirus infection were also analyzed.

**Results:**

There was no significant difference in sex and age among the four groups (P>0.05). However, there was a significant difference in the frequency of diarrhea and vomiting (p<0.05). In addition, there were significant differences in creatine kinase, CK-MB, AST and ALT levels in children with metabolic acidosis of different severities.

**Conclusion:**

With the aggravation of metabolic acidosis, infectious diarrhea caused by rotavirus is characterized by the aggravation of hepatic function and myocardial cells.

## INTRODUCTION

As an RNA virus, rotavirus falls into the category of enteroviruses and is transmitted mainly by the fecal-oral and respiratory routes. Rotavirus infection may not only lead to absorptive dyspepsia in pediatric patients but also cause clinical symptoms, such as diarrhea, emesis and fever. In severe cases, secondary onset of dehydration and electrolyte disorders takes place, which is also accompanied by acidosis at different levels. Throughout the world, especially in low-/middle-income countries, rotavirus is a major infectious agent of acute gastroenteritis in children, accounting for approximately 50% of all etiological factors of gastroenteritis.^1^-^3^ It cannot only cause intestinal infection but also cause organ damage. In recent years, an increasing number of cases of rotavirus enteritis with myocardial, liver and kidney damage have been found.^4^ This paper focuses on the relationship between myocardial enzymes, liver function and metabolic acidosis in children with rotavirus infection diarrhea.

## METHODS

*Seventy* Pediatric patients (33 males and 37 females, average age: 6.75±2.74 months) who sought medical care in Baoding Children’s Hospital from October 2017 to April 2018 were retrospectively studied. Pediatric patients with diarrhea and other clinical symptoms and confirmed rotavirus infection were selected as the research objects. According to the clinical features of biochemical indicators and mental states, the patients were divided into four groups: an acidosis-free group, a mild acidosis group, a moderate acidosis group and a severe acidosis group.

The study was approved by the Institutional Ethics Committee of Baoding Children’s Hospital on January 10, 2020, and written informed consent was obtained from all participants.

### Inclusion criteria

1. Rotavirus antigen was detected as positive by a colloidal gold method for feces.
2. Clinical signs such as diarrhea were manifest.
3. No congenital diseases, such as heart, hepatic or gall bladder diseases, were present.

### Exclusion criteria

1. Rotavirus in feces was negative.
2. No clinical signs such as diarrhea were observed.
3. Congenital diseases, such as heart, hepatic and gall bladder diseases, were diagnosed.

### Research methodology

1. ***Clinical observation indexes:*** Sex, age, number of episodes of diarrhea and emesis, etc., were recorded.
2. ***Rotavirus detection:*** RV antigens of feces taken from pediatric patients were tested by a colloidal gold method. The corresponding reagent was provided by Shenzhen Huian Bioscience Technology Co., Ltd. To be specific, approximately 100 mg of fecal samples were taken from each child patient and then placed in bottles of diluent; after stirring and uniformly blending, 3 drops of fecal supernatant were added into the bottle that was then put at an ambient temperature for 10 minutes. After that, the corresponding results were interpreted. All fecal sample detection was completed within 1 hour after sampling. If a red control line and a red quality control line simultaneously appeared in a test card, rotavirus was detected as positive; if only a red quality control line appeared, it was detected as negative; that is, no rotavirus was found in the specimen. If no red quality control line appeared on the test card, the detection was judged as invalid, in which case, the corresponding specimen was tested again.
  Based on a single-blind technique, all procedures were performed by a specially assigned person in accordance with the *National Guide to Clinical Laboratory Procedures (4^th^ Edition)* and specifications of the test consumables.
3. ***Laboratory observation indexes:*** Stomachs of pediatric patients were held empty for at least six hours. In the morning, 2 ml of venous blood was sampled and placed in a tube. Subsequent to centrifugation, the supernatant was obtained. Here, an Roche fully automatic biochemical analyzer was utilized. The following indexes of pediatric patients were recorded:
  - CK, normal value range: 25-200 U/L.
  - CK-MB, normal value range: 0-25 U/L.
  - ALT, normal value range: 9-50 U/L.
  - AST, normal value range: 15-40 U/L.
4. ***Metabolic acidosis:*** Metabolic acidosis severity was identified in line with pathogenetic conditions, such as arterial blood gas analysis, biochemical detection, mental state and skin mucous membrane color of pediatric patients.

Indicators and symptoms of patients in the acidosis-free group can be described as follows: pH: 7.35~7.45; HCO3^–^ (mmol/L): 22~27; normal mental status; normal respiration; and normal oral mucous membrane color. For the mild acidosis group, their indicators and symptoms were as follows: pH: 7.30~7.35; HCO3^–^ (mmol/L): 18~22; normal mental status; breathing slightly fast; and normal oral mucous membrane color. Regarding the moderate acidosis group, their indicators and symptoms were as follows: pH: 7.25~7.29; HCO3- (mmol/L): 14~18; reduced activity; dysphoria; breathing deeply; and deep red mucous membrane color. In terms of the severe acidosis group, the indicators and symptoms were as follows: pH: <7.25; HCO3^–^ (mmol/L): <14; reduced activity; dysphoria or lethargy; breathing deeply and fast; and mucous membrane cyanosis.

### Statistical analysis

SPSS 21.0 was selected to carry out statistical analysis. Normally distributed measurement data are expressed as the mean ± standard deviation (χ±s). For intergroup measurement data, a normality test was first conducted, and variance analysis was performed on data conforming to a normal distribution. In addition, intergroup data statistics are represented by the number of use cases, and their comparison was performed by means of the chi-square test. p<0.05 was used as the significance threshold.

## RESULTS

In total, 70 pediatric patients with diarrhea from rotavirus infection were included. In detail, the acidosis-free group consisted of 26 cases (12 male and 14 female patients; average age: 7.07±2.96 months), and the average frequencies of diarrhea and emesis were 4.11±1.75 and 2.96±1.34, respectively. In the mild acidosis group, there were 17 cases (8 male and 9 female patients; average age: 6.00±2.47 months); the average frequencies of diarrhea and emesis were 4.47±1.80 and 4.29±2.05, respectively. Moreover, 10 cases were included in the moderate acidosis group, involving four male and six female patients (average age: 6.30±2.49), and the average frequencies of diarrhea and emesis were 5.70±1.56 and 5.60±1.17, respectively. Finally, there were 17 cases (9 male and 8 female patients; average age: 7.29±2.80 months) in the severe acidosis group, and the average frequencies of diarrhea and emesis were 8.94±1.47 and 6.82±1.62, respectively. Differences in both age (*F*=0.889; *P*>0.05) and sex (*P*>0.05) of pediatric patients suffering from different levels of acidosis showed no statistical significance. However, differences in the frequencies of diarrhea (*F*=31.814; *P*<0.01) and emesis (*F*=21.74; *P*<0.01) were statistically significant, as shown in Table-I.

**Table-I:** General conditions of 70 pediatric patients.

	No. of cases	Sex (M/F)	Age (in months)	Diarrhea (No. of episodes)	Emesis (No. of episodes)
Acidosis-free group	26	12/14	7.07±2.96	4.11±1.75^a^	2.96±1.34^a^
Mild acidosis group	17	8/9	6.00±2.47	4.47±1.80^a^	4.29±2.05^b^
Moderate acidosis group	10	4/6	6.30±2.49	5.70±1.56^b^	5.60±1.17^c^
Severe acidosis group	17	9/8	7.29±2.80	8.94±1.47^c^	6.82±1.62^d^
Statistics		χ^2=^0.444	F=0.889	F=31.814	F=21.74
P-value		>0.05	>0.05	<0.01	<0.01

***Notes:*** Among patients in the four groups, if a superscript of the number of episodes of diarrhea is the same as that of the number of episodes of emesis, it signifies that no statistical significance was found for the differences; otherwise, it means that their differences are statistically significant.

On average, hepatic functions and myocardial enzyme levels of pediatric patients in four groups can be detailed as follows: CK: 138.23±41.93 U/L, CK-MB: 21.10±6.81 U/L, AST: 33.61±10.75 U/L and ALT: 41.73±12.07 U/L for those in the acidosis-free group; CK: 208.08±18.85 U/L, CK-MB: 48.37±11.25 U/L, AST: 52.00±14.05 U/L and ALT: 64.23±17.28 U/L for the mild acidosis group; CK: 257.81±15.15 U/L, CK-MB: 65.16±10.64 U/L, AST: 63.70±12.60 U/L and ALT: 76.50±13.62 U/L for the moderate acidosis group; and CK: 268.74±14.01 U/L, CK-MB: 66.58±15.81 U/L, AST: 76.76±9.76 U/L and ALT: 100.29±26.62 U/L for the severe acidosis group. Additionally, differences in CK (*F=*85.37; *P*<0.01), CK-MB (*F=*72.41; *P*<0.01), AST (*F=*50.15; *P*<0.01) and ALT (*F=*37.40; *P*<0.01) levels were all statistically significant in the four groups of pediatric patients suffering from acidosis at various levels of severity, as shown in Table-II.

**Table-II:** Hepatic functions and myocardial enzyme levels of 70 pediatric patients.

	No. of cases	CK (U/L)	CK-MB (U/L)	AST (U/L)	ALT (U/L)
Acidosis-free group	26	138.23±41.93^a^	21.10±6.81^a^	33.61±10.75^a^	41.73±12.07^a^
Mild acidosis group	17	208.08±18.85^b^	48.37±11.25^b^	52.00±14.05^b^	64.23±17.28^b^
Moderate acidosis group	10	257.81±15.15^c^	65.16±10.64^c^	63.70±12.60^c^	76.50±13.62^b^
Severe acidosis group	17	268.74±14.01^c^	66.58±15.81^c^	76.76±9.76^d^	100.29±26.62^c^
*F*-value		85.37	72.41	50.15	37.40
*P*-value		<0.01	<0.01	<0.01	<0.01

***Notes:*** Among patients in the four groups, if the superscripts of observation indexes are the same, it signifies that no statistical significance was found for their differences; otherwise, it means that their differences are statistically significant.

## DISCUSSION

Rotavirus infection is the most common pathogenesis of infectious diarrhea in pediatrics, and its incidence rate in patients aged 3~24 months reaches its peak.^5^ Rotaviruses are divided into G and P genotypes based on two genomic segments’ nucleotide sequences, VP7 and VP4, respectively. Currently, 27 G and 37 P types have been described.^6^ Symptoms of rotavirus infection include emesis, fever and diarrhea. Specifically, diarrhea caused by rotavirus belongs to nonbloody diarrhea, which is distinct from bacterial infectious diarrhea.^7^ Clinical symptoms of pediatric patients with diarrhea from rotavirus infection are more serious than those of pediatric patients without rotavirus infection, which are manifest as more frequent emesis and diarrhea; in severe cases, the number of episodes of diarrhea reaches at least 10 on a daily basis. Rotavirus is the most common infectious diarrhea agent and can cause severe dehydration, electrolyte imbalance and even death in young children. For many years, rotavirus (RV) pathology has remained an undervalued condition and is limited only to the gastrointestinal tract in the eyes of most clinicians. For these reasons, when compared with diarrhea of other causes, diarrhea from rotavirus infection is much more likely to result in dehydration, electrolyte disturbances and metabolic acidosis among pediatric patients. As reported, dehydration levels are positively correlated with degrees of hepatic function abnormality.^8^ In this research, it was also found that once metabolic acidosis becomes more severe, significant differences lie in AST and ALT levels compared with those of patients free of metabolic acidosis.

The pathogenesis of diarrhea from rotavirus infection is still not clarified. However, recent evidence from hidden systemic implications of RV infection has renewed interest in this pathology. RV pathology is systemic; the RV goes beyond the intestinal lumen irrespective of the presence or absence of diarrhea.^9^ Generally, patients suffering from diarrhea from rotavirus infection show an insignificant rise in their C-reactive protein level and white blood cell count. In comparison with other virus and bacterial infections, rotavirus infection is less likely to induce pulmonary symptoms. Therefore, there are two possible mechanisms of diarrhea from rotavirus infection. One is malabsorption secondary to osmotic diarrhea that is induced by enterocyte damage, death, or epithelial absorption degradation, and the other is secretory diarrhea caused by nonstructural proteins, such as NSP4.^10^ In addition, 5-HT secretion mediated by rotavirus infection may activate signaling pathways that give rise to diarrhea and emesis, and it is also involved in the onset of diarrhea and emesis among pediatric patients.^11^

According to research findings, rotavirus that causes intestinal infection can also be located to extraintestinal tissues (e.g., heart, liver and central nervous system) by virtue of RNA reverse transcriptase (RT) to cause viral infections of associated tissues; in severe cases, tissue damage may also be incurred.^12^-^15^ The function of the intestinal mucosal barrier in infants is poor. The virus can enter the lymph circulation and blood circulation of the body through the intestinal mucosal barrier, which causes infection of extraintestinal organs and immaturity of intestinal immune function in infants. In particular, the lack of secretory IgA is another reason that the virus enters the blood circulation through the intestinal mucosa. In addition, the immaturity of the heart, liver, kidney and other organs in infants are all the causes of damage.^16^ Here, hepatic function indexes (AST & ALT) and myocardial enzymes (CK & CK-MB) of pediatric patients with rotavirus infections are proven to significantly increase along with the aggravation of patients’ conditions. It has also been indirectly demonstrated by these results that aggravation of rotavirus infections leads to damage to extraintestinal tissues in simple ways.^17^

There are two reasons for the rise in myocardial enzyme levels in patients with rotavirus infection caused by metabolic acidosis.^18^ First, cardiac muscle cells are directly attacked by toxins produced by rotavirus. Second, dehydration caused by frequent diarrhea and emesis of pediatric patients leads to secondary effective circulating blood volume reduction. Therefore, myocardial cells in pediatric patients are in a state of high carbon dioxide and hypoxia, and metabolic acidosis occurs, which produces more metabolites and oxygen free radicals, including prostaglandin and interleukin-6, and causes cell membrane and mitochondrial damage.^19^ Eventually, cardiac muscle cells are damaged.

Clinically, the degree of acidosis should be judged according to the biochemical indexes and the state of consciousness of pediatric patients with rotavirus; the damage of extraintestinal tissues and organs involves liver function and myocardial cells; rotavirus infection caused by diarrhea should be detected as soon as possible. Chronic metabolic acidosis with hyperuricemia in patients with rotavirus gastroenteritis can lead to acute obstructive uropathy and the formation of ammonium urate calculus. Therefore, the normalization of metabolic acidosis is very important to prevent the onset of obstructive uropathy associated with rotavirus gastroenteritis.^20^

### Limitations of the study

First, this study was a retrospective case-control study, which was approved by the hospital ethics committee and obtained patient consent, but did not receive an RCT number. Second, there is a certain subjectivity in the grouping of patients, and the grouping criteria should be more objective in future research.

## CONCLUSION

With the aggravation of metabolic acidosis, infectious diarrhea caused by rotavirus is characterized by the aggravation of hepatic function and myocardial cells. Additionally, it is suggested that when clinical treatment of infectious diarrhea, pediatricians should also pay attention to the damage of extraintestinal tissues and organs, and carry out relevant examination in time to avoid aggravation of the disease.

### Authors’ Contributions:

**NYZ and YDZ** designed this study, prepared this manuscript, and are responsible for and accountable for the accuracy or integrity of the work. **QWD** collected and analyzed the clinical data. **LH** significantly revised this manuscript.
